# Evaluation and comparison of spatial cluster detection methods for improved decision making of disease surveillance: a case study of national dengue surveillance in Thailand

**DOI:** 10.1186/s12874-023-02135-9

**Published:** 2024-01-19

**Authors:** Chawarat Rotejanaprasert, Kawin Chinpong, Andrew B. Lawson, Peerut Chienwichai, Richard J. Maude

**Affiliations:** 1 Department of Tropical Hygiene, Faculty of Tropical Medicine, Mahidol University, Bangkok, Thailand; 2 Mahidol-Oxford Tropical Medicine Research Unit, Faculty of Tropical Medicine, Mahidol University, Bangkok, Thailand; 3 Chulabhorn Learning and Research Centre, Chulabhorn Royal Academy, Bangkok, Thailand; 4 Department of Public Health Sciences, Medical University of South Carolina, Charleston, SC USA; 5 Usher Institute, University of Edinburgh, Edinburgh, UK; 6 Princess Srisavangavadhana College of Medicine, Chulabhorn Royal Academy, Bangkok, Thailand; 7 Harvard T.H. Chan School of Public Health, Harvard University, Cambridge, MA USA; 8 Centre for Tropical Medicine and Global Health, Nuffield Department of Medicine, University of Oxford, Oxford, UK; 9 The Open University, Milton Keynes, UK

**Keywords:** Spatial, Cluster detection, Dengue, Surveillance, Thailand

## Abstract

**Background:**

Dengue is a mosquito-borne disease that causes over 300 million infections worldwide each year with no specific treatment available. Effective surveillance systems are needed for outbreak detection and resource allocation. Spatial cluster detection methods are commonly used, but no general guidance exists on the most appropriate method for dengue surveillance. Therefore, a comprehensive study is needed to assess different methods and provide guidance for dengue surveillance programs.

**Methods:**

To evaluate the effectiveness of different cluster detection methods for dengue surveillance, we selected and assessed commonly used methods: Getis Ord ${G}_{i}^{*}$, Local Moran, SaTScan, and Bayesian modeling. We conducted a simulation study to compare their performance in detecting clusters, and applied all methods to a case study of dengue surveillance in Thailand in 2019 to further evaluate their practical utility.

**Results:**

In the simulation study, Getis Ord ${G}_{i}^{*}$ and Local Moran had similar performance, with most misdetections occurring at cluster boundaries and isolated hotspots. SaTScan showed better precision but was less effective at detecting inner outliers, although it performed well on large outbreaks. Bayesian convolution modeling had the highest overall precision in the simulation study. In the dengue case study in Thailand, Getis Ord ${G}_{i}^{*}$ and Local Moran missed most disease clusters, while SaTScan was mostly able to detect a large cluster. Bayesian disease mapping seemed to be the most effective, with adaptive detection of irregularly shaped disease anomalies.

**Conclusions:**

Bayesian modeling showed to be the most effective method, demonstrating the best accuracy in adaptively identifying irregularly shaped disease anomalies. In contrast, SaTScan excelled in detecting large outbreaks and regular forms. This study provides empirical evidence for the selection of appropriate tools for dengue surveillance in Thailand, with potential applicability to other disease control programs in similar settings.

**Supplementary Information:**

The online version contains supplementary material available at 10.1186/s12874-023-02135-9.

## Background

Dengue is one of the most prevalent mosquito-borne diseases in humans with an approximate global burden of 390 million annual infections, of which 96 million are symptomatic [1]. The infection is caused by dengue virus mainly transmitted by *Aedes* mosquitoes which are commonly found in tropical and sub-tropical regions and having four serotypes, DENV-1 to 4. Dengue particularly prevalent in South-East Asia with the reported incidence in Thailand being among the highest in the world [2]. Dengue poses a substantial burden for the healthcare system and financially impacts households of Thailand [3, 4] with approximately 100,000 annual cases reported to the Thai Ministry of Public Health [5]. It is endemic and causes epidemics every few years [6].

The two available vaccines for dengue have around 80% short-term efficacy preventing symptomatic disease. Dengvaxia can only be administered to people who have previously been infected, requiring pre-vaccination antibody screening, [7] whereas Qdenga can be given regardless of prior exposure but efficacy is lower against serotypes DENV-1 and 3 [8]. With a lack of specific treatment, public health interventions targeted against mosquitoes have remained a focus of dengue control to interrupt the transmission. Rapid detection of outbreaks is an important goal to effectively allocate prevention and control resources. Efficient and reliable surveillance systems are then crucial especially for endemic countries. Spatial cluster detection can identify areas of elevated risk and can facilitate policy decision making and budget allocation of limited public health resources in Thailand.

Different methods have been proposed to locate and identify disease clusters dependent on whether the locations of the clusters are known (focused) or unknown (non-focused). Models for focused clusters are designed for detecting preconceived patterns linked to objects or putative sources [9, 10]. Models for non-focused clusters, on the other hand, are designed to estimate the relative risk for each area within the study area. Typically, these models accommodate extra spatial variability in different ways (for example [11–15]). Simulation studies have been used to evaluate and compare spatial detection methods due to the ability to understand the behavior of statistical methods as parameters of interest can be set to be known values from the process of generating the data [16]. Thus, this process allows us to investigate and better understand properties of methods using ground-truth scenarios.

In recent decades a range of computing approaches have been introduced and utilized extensively to examine dengue clusters (see examples [17–21]). They usually aim to determine whether a disease count exceeds an expected value obtained based on an overall population. Clustering tests are distinguished according to whether they are global (aiming to assess the general existence of clusters) or local (aiming to identify individual clusters). For disease surveillance, local cluster detection however might be more useful for disease control activities and planning as it helps to identify and prioritize high-risk areas. Despite the wide range of applications, there is no general guidance as to which is the most appropriate cluster detection method for dengue spatial surveillance. The patterns or shapes of disease clusters may vary due to various risk factors, and it is likely that some methods are more suited to detect specific cluster morphologies than others. Therefore, there is a need to conduct a comprehensive study to compare local spatial cluster detection methods to better understand their operating characteristics and provide a general guide for dengue surveillance programs.

Hence, the purpose of this article is to evaluate the performance of widely used methods for dengue cluster detection. Four methods were selected for different types of spatial cluster detection methods based on previous reviews [20, 21]. The aim was to extensively study each method’s behavior for different disease characteristics including cluster shape, risk level between high-risk and normal risk areas, and testing performance measures of spatial clusters. A simulation study was conducted to examine those methods which differ in the way they detect clusters. To thoroughly investigate the cluster detection in practice, all four methods were further applied to a case study of dengue surveillance in Thailand in 2019 to explore their performance in a real setting. Thus, our comprehensive comparison study can be a useful guide for choosing appropriate spatial anomaly detection methods to identify and target clusters and increase efficiency of dengue control programs.

## Methods

The four spatial cluster detection methods used in the comparison were Getis Ord ${G}_{i}^{*}$, Moran’s I, SaTScan and Bayesian disease mapping via exceedance probability. The first three techniques are testing-based methods whereas Bayesian disease mapping is a model-based procedure. These are described in detail below. After that, the setting of the simulation study is explained including the data generation mechanism and the parameter specification is described and performance evaluation metrics used for the comparison in this study are provided.

### Cluster detection methods

#### Getis Ord ${G}_{i}^{*}$

The first method considered in this study was Getis Ord ${G}_{i}^{*}$ [22]. This approach can be used to indicate potential clusters by looking at each spatial unit within the context of neighboring areas. To be a statistically significant hot spot, an area needs to have a high value and be surrounded by other areas with high values. The local sum for a unit and its neighbors is compared proportionally to the sum of all features; when the local sum is very different from the expected local sum, and when that difference is too large to be the result of random chance, a statistically significant z-score results.

Let $D$ be the study area (map) and ${y}_{i}\in D$ represents values at node or spatial unit $i$ in the study area $D$. The standardized form of Getis Ord ${G}_{i}^{*}$ at spatial unit $i$ can be expressed as

1 $${G}_{i}^{*}=\frac{\left(\sum_{j=1}^{N}{w}_{ij}{y}_{j}-\overline{Y }\sum_{j=1}^{N}{w}_{ij}\right)}{SD\sqrt{\frac{\left(N\sum_{j=1}^{N}{w}_{ij}^{2}\right)-{\left(\sum_{j=1}^{N}{w}_{ij}\right)}^{2}}{N-1}}} ;\forall j$$

where

$$\begin{array}{c}\overline{Y }=\frac{1}{N}\sum\limits_{j=1}^{N}{y}_{j}\\ SD=\sqrt{\frac{\sum_{j=1}^{N}{y}_{j}^{2}}{N}-{\overline{Y} }^{2}}\\ i,j\in \left\{n \right| 0\le n<N; n,N\in {\mathbb{N}}\}.\end{array}$$

$N$ denotes the number of areas in the map $D$. $S$ is standard deviation of all values ${y}_{i}$ in map $D$. ${w}_{ij}\in W$ represents spatial weight in the spatial matrix $W$, and $\overline{Y }$ indicates average of all values ${y}_{i}$ in space $D$. The spatial units with significantly high ${G}_{i}^{*}$ values are identified as a hotspot cluster or high-value group whereas units with low values of ${G}_{i}^{*}$ can be conversely defined as a cold spot cluster. This calculation relies on hypothesis testing under the null hypothesis of spatial independence for which significance can be detected using the z-scores and *p*-values from a permutation distribution.

#### Anselin local Moran’s I (Local Moran)

Another widely used cluster detection technique is the Anselin local Moran’s I which is a localized analogy of the global Moran’s I [23]. The Anselin local Moran’s I statistic ${I}_{i}$ for area $i$ can be written as

2 $${I}_{i}=\frac{\sum_{j=1}^{N}{w}_{ij}\left({y}_{i}-\overline{Y }\right)\left({y}_{j}-\overline{Y }\right)}{\frac{1}{N}\sum_{j=1}^{N}\left({y}_{j}-\overline{Y }\right)};\forall j\ne i$$

with

$$\begin{array}{c}\overline{Y }=\frac{1}{N}\sum\limits_{i=1}^{N}{y}_{i}\\ i,j\in \left\{n \right| 0\le n<N; n,N\in {\mathbb{N}}\}\end{array}$$

where ${y}_{i}$ and ${y}_{j}$ stand for values of node $i$ and $j$ respectively in the study area (map) $D$ and $\overline{Y }$ represents the global average of node values in the map.

A positive value for *I* indicates that a feature has similar neighboring values which can be a high or low cluster. A negative value for *I* indicates that a feature has neighboring features with dissimilar values, called an outlier. Thus, this method classifies each significant spatial unit into 4 types according to quadrant plots: 1) higher unit values with higher spatial lags (denoted as cluster HH), 2) lower node values with higher lags (denoted as outlier LH), 3) lower unit values with lower lags (denoted as cluster LL), and 4) higher node values with lower spatial lag values (denoted as outlier HL). However, in any case, the *p*-value for the feature must be small enough for the cluster or outlier to be considered statistically significant. Note that this overall calculation is also called Local Indicator of Spatial Association or LISA.

#### Spatial scan statistics (SaTScan)

Spatial scan statistics is a popular method that has been used in spatial analysis [24, 25]. The step of discrete spatial scan in the SaTScan procedure which was adapted in this study starts from defining centroids of every aggregated area, which were used as representative of location at node $i$. After that, circle zones were applied at every node in spatial space, then a likelihood ratio statistic was computed depending on the numbers of observed and expected values within and outside the circles and compared likelihood function under the null hypothesis. On the contrary, an alternative hypothesis was served under a Poisson distribution. For a specific scanning window, the likelihood function is proportional to

3 $${\left(\frac{\gamma }{E\left(\gamma \right)}\right)}^{\gamma }{\left(\frac{C-\gamma }{C-E\left(\gamma \right)}\right)}^{C-\gamma }I\left(\gamma ,E\left(\gamma \right)\right)$$

with

$$I\left(\gamma ,E\left(\gamma \right)\right)=\left\{\begin{array}{c}1;\gamma >E\left(\gamma \right)\\ 0;otherwise.\end{array}\right.$$

$\gamma$ and $E\left(\gamma \right)$ respectively denote the observed number and expected value of cases within the specific circular window. $C$ represents the total number of cases occurring in the study $D$, and $I\left(\gamma ,E\left(\gamma \right)\right)$ is the indicator function which equals 1 if the observed number is larger than the expected and equal to 0 otherwise. The point estimates are usually calculated at which the likelihood function is maximized over all circle locations and sizes. In this study, the *p*-value was approximated by Monte Carlo hypothesis testing which compared the rank of the maximum likelihood between the data in the original and simulated spaces. Significant hotspots were then identified as all centroids within significant point estimates in the scanning window.

#### Bayesian disease mapping via exceedance probability

The fundamental feature of Bayesian disease mapping is the use of probability for measuring uncertainty in statistical inference. The major appeal of this approach is in considering uncertainty in the predictions or estimates and the straightforward incorporation of spatial structure as prior distributions which is very useful especially in spatial epidemiology [26]. There are two main spatial models in areal Bayesian disease mapping: Besag and Besag-York- Molliè (BYM) models. These specifications are widely used in hierarchical Bayesian models operated using random effects with unstructured and spatially structured heterogeneity [27]. For the $i$ th area under spatial space $D$, the Bayesian model for the number of cases ${y}_{i}$ is usually assumed to follow a Poisson likelihood as [28, 29]

7 $${y}_{i}\sim Poisson\left({e}_{i}{\theta }_{i}\right)$$

where ${e}_{i}$ denotes the expected value while ${\theta }_{i}$ represents the incidence ratio relative to that expected at area $i$. The model disintegrates logarithmic incidence ratio into summation of unstructured and structured random effects. In the BYM model, the random effects are linked to the relative ratio as

8 $${\text{log}}\left({\theta }_{i}\right)={b}_{0}+{u}_{i}+{v}_{i}.$$

On the other hand, the Besag model, which contains less parameters than BYM, is defined as

9 $${\text{log}}\left({\theta }_{i}\right)={b}_{0}+{u}_{i}.$$

${b}_{0}$ is the overall intercept. ${u}_{i}$ and ${v}_{i}$ respectively represent the spatially structured and unstructured error terms. The model for spatial effect is often defined using an intrinsic conditional autoregressive model (iCAR) prior distribution on the set of neighborhoods of the $i$ th node, ${\delta }_{i}$, as

10 $${u}_{i}|{{\varvec{u}}}_{-i}\sim Normal\left(\frac{1}{\left||{\delta }_{i}\right||}\sum_{j\in {\delta }_{i}}{u}_{j},{s}_{i}^{2}\right)$$

where ${s}_{i}^{2}$ represents its variance parameter. The overall intercept,${b}_{0}$, and unstructured random effect,${v}_{i}$, is usually assumed to be a zero-mean normal distribution [30, 31]. All precision parameters were set as the default distribution as Log-Gamma (1, 0.00005). The statistical inference in Bayesian settings is traditionally based on sampling procedures, for example, the Markov Chain Monte Carlo (MCMC). Unfortunately, the posterior function from Bayesian methods is often committed to a high-dimensional integration form which is not typically tractable in a closed-form [32]. In addition, the MCMC procedure can be too computationally intensive and slow for infectious disease surveillance focusing on timeliness [33, 34]. The Integrated Nested Laplace Approximation (INLA) which requires less computing resources can be an alternative [35]. Thus, the posterior estimates in this study were computed using INLA. An anomaly can be identified using the exceedance probabilities as ${\text{Pr}}\left({\theta }_{i}>q\right)$ where *q* is the threshold. When* q* = 1, the expected rate or baseline was used as the threshold. In this study, a hotspot was defined as a location with ${\text{Pr}}\left({\theta }_{i}>q\right)$ greater than a cut-off point, i.e. ${\text{Pr}}\left({\theta }_{i}>q\right)>1-\alpha$ where $\alpha$ was the pre-specified level of significance.

### Simulation study

#### Simulated scenarios and data generation

**Fig. 1:**
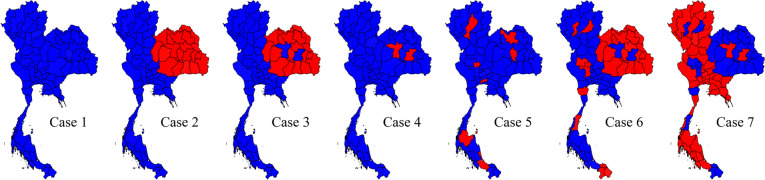
Seven designed scenarios of the simulation study. The hotspot areas are red while blue areas represent non-cluster areas

The map of all 77 provinces in Thailand was used as the base map in our simulation study. The geographic coordinates of the province centroids and province boundaries were from the GEO package file in the database of Global Administrative Areas, a high-resolution database of country administrative areas. In each replication *k* = 1, …, *K*, the number of dengue cases in province *i* was generated from a Poisson distribution as ${y}_{ik}\sim Poisson\left({e}_{ik}{\theta }_{ik}\right)$ where ${e}_{ik}$, and ${\theta }_{ik}$ were respectively expected rate and relative risk. The provincial expected rate was set as ${e}_{i}=\frac{\sum_{i}{y}_{i}}{\sum_{i}{pop}_{i}}\times {pop}_{i}$ where, here, *y*_*i*_ and *pop*_*i*_ were the number of dengue cases and population for each province in 2019, collated from the surveillance reporting system, Bureau of Epidemiology, Department of Disease Control, Ministry of Public Health (MOPH). All the cases were aggregated and notified in 2019 at provincial level. The relative risk, ${\theta }_{ik}$, was assumed to be the designated value of 2 representing hotspot clusters, double the expected rate (red areas in Fig. 1), while the rest were set to 1 as the baseline level (blue areas in Fig. 1).

To comprehensively address various surveillance and control situations, seven distinct scenarios were formulated, as depicted in Fig. 1. Scenario case 1 served as a null scenario, replicating a situation devoid of any outbreaks. Scenario case 2 represented an uncontrollable outbreak characterized by a large disease cluster, while case 3 mirrored scenario case 2, but with specific areas intentionally left under-controlled to assess the adaptability of cluster detection methods. In contrast to the large outbreak, scenario cases 4 and 5 were crafted to feature isolated disease hotspots, with case 5 exhibiting a more widespread disease distribution across the map. To further assess disease anomalies with diverse conditions and shapes, scenarios 6 and 7 were created by combining elements from other cases, providing a comprehensive representation of various disease anomaly scenarios. These scenarios aimed to replicate a wide range of situations encountered in disease surveillance, encompassing varying spatial shapes and conditions.

#### Method and model specification

Based on the cluster detection procedures that were described in the previous section, it was necessary to specify a spatial weight matrix. There are a number of contiguity matrices, however the first order neighborhood structure was appropriate for Thai provincial-level [11] and was used as the base map in our simulation and case studies. More details about the spatial contiguity matrix are provided in section S1 in the supplementary document. The Database of Global Administrative Areas (GADM) was managed using GeoPandas 0.10.2 for file loading [36] and libpysal 4.6.2 for weight calculation [37]. For the simulated iteration $k$, Getis Ord Gi* and Local Moran’s I were performed on the standardized morbidity ratio (SMR) for *i*th province as *SMR*_i_ = *y*_i_/*e*_i_ using library esda 2.4.1 within Python version 3.10.4 [37]. Outputs of Local Moran’s I for same iteration $k$ were classified into 2 versions, only HH labels as hotspots (further annotated the procedure in the results as “Local Moran1”, and version of hotspots including HH and diamond-outlier HL, named as “Local Moran2”).

The cluster detection of SaTScan was computed using command-line interface version 10.0.2 combined with rsatscan library version 0.3.9200 [38]. SaTScan was applied using the Poisson model with circular window including the limitation of circle size in population at risk, and size in max circle of population that did not exceed 50%. Centroid nodes within the significant windows were interpreted as hotspots. For Bayesian disease mapping, the Besag and BYM models were implemented using R-INLA library version 22.05.07 [39]. SaTScan and Bayesian models were implemented in R version 4.2. The clusters from Bayesian modeling were defined using exceedance probability as ${\text{Pr}}\left({\theta }_{i}>{\theta }^{*}\right)>$ $1-\alpha$. The threshold ${\theta }^{*}$ is usually defined as a baseline or endemicity [40]; here the threshold ${\theta }^{*}$ was chosen to be 1 to represent the null situation. The significance level was specified as $\alpha$ = 0.05 in this study.

#### Evaluation of spatial clustering procedures

Surveillance systems for infectious diseases have to balance the outbreak detection accuracy against disease control resources. So, the concepts of optimal criteria, accuracy, sensitivity, specificity, positive predictive value (PPV), and negative predictive value (NPV), are useful to compare and assess validity of cluster detection methods. These five evaluation metrics were adopted for method comparison and performance evaluation in this simulation study. The notation used in calculation was as follows. $TP$ = true positive, $FP$ = false positive, $FN$ = false negative, and $TN$ = true negative. Accuracy, summation of true positive and true negative over total counts, was calculated as $\frac{TP+TN}{TP+FP+FN+TN}$. Sensitivity, the probability of a positive test given the existence of hotspots, was defined as $\frac{TP}{TP+FN}$ while specificity, the proportion of negative tests among the non-hotspots, was computed as $\frac{TN}{FP+TN}$. Sensitivity and specificity are normally applied to evaluate the ability of a test to detect or to rule out correctly with the ground truth condition [41]. On the other hand, PPV and NPV yield the probability for appearance (or inapparency) of hotspots based on test results [42]. PPV was calculated as $\frac{TP}{TP+FP}$ while NPV was computed as $\frac{TN}{FN+TN}$. These indicators were spatially visualized at provincial level to compare and discuss the performance of the models thereafter.

## Results

### Simulation study

To demonstrate empirical evidence of the detection method comparison, a simulation study with different ground-truth scenarios was conducted. Due to limited space in the main text, map results of scenario cases 1–5 are provided in the supplementary document S2 as Figures S1-S5. Based on 200 simulated datasets (*K* = 200), all methods performed fairly well in the first scenario as the null situation for the whole map while Getis Ord ${G}_{i}^{*}$ had the lowest accuracy of 0.95 in this scenario (Figure S1). The results of case scenario 2 are shown in Figure S2 representing a large outbreak cluster in the northeast. The methods with the lowest accuracy were Getis Ord ${G}_{i}^{*}$ and Local Moran with the worst sensitivity and PPV along the outbreak boundary. SaTScan had the highest accuracy, slightly better than Bayesian mapping with a lower specificity. The third case scenario was similar to the second except for two non-cluster provinces in the middle representing effective public health interventions. Interestingly, only local Moran’s I could detect the isolated hotspots in the middle of a large outbreak as seen in the specificity map in Figure S3. The Getis Ord ${G}_{i}^{*}$ had the overall worst performance for this scenario while SaTScan and Bayesian mapping yielded a false alarm over the two non-cluster provinces. Nonetheless, there was a boundary issue with Getis Ord ${G}_{i}^{*}$ and Local Moran as seen in low sensitivity and PPV for those methods in Figure S3 and Table 1. Overall SaTScan and Bayesian mapping were the best in this case scenario.

**Table 1:** Evaluation measures of the all cluster detection methods under different scenarios

Scenario	Detection method	Evaluation metric				
		Accuracy	Sensitivity	Specificity	PPV	NPV
1	Getis Ord ${G}_{i}^{*}$	0.95039	-	0.95039	-	1
	Local Moran1	0.976169	-	0.976169	-	1
	Local Moran2	0.951429	-	0.951429	-	1
	SaTScan	0.992727	-	0.992727	-	1
	BESAG (q = 1)	0.970584	-	0.970584	-	0.995
	BYM (q = 1)	0.996429	-	0.996429	-	1
2	Getis Ord ${G}_{i}^{*}$	0.970519	0.891905	1	1	0.96111
	Local Moran1	0.97013	0.890476	1	1	0.960633
	Local Moran2	0.967662	0.890476	0.996607	0.990401	0.9605
	SaTScan	0.999351	0.997857	0.999911	0.999773	0.999211
	BESAG (q = 1)	0.962792	1	0.948839	0.885957	1
	BYM (q = 1)	0.964091	1	0.950625	0.889646	1
3	Getis Ord ${G}_{i}^{*}$	0.911234	0.745789	0.965431	0.875372	0.920933
	Local Moran1	0.936753	0.745789	0.99931	0.997508	0.923417
	Local Moran2	0.933831	0.745789	0.995431	0.982776	0.923133
	SaTScan	0.973766	0.998947	0.965517	0.904667	0.999649
	BESAG (q = 1)	0.945065	1	0.927069	0.821923	1
	BYM (q = 1)	0.950714	0.999737	0.934655	0.838143	0.999909
4	Getis Ord ${G}_{i}^{*}$	0.894675	0.0425	0.9174	0.013774	0.972912
	Local Moran1	0.942532	0	0.967667	0	0.97317
	Local Moran2	0.926818	0.06	0.949933	0.043964	0.974267
	SaTScan	0.98474	0.9525	0.9856	0.701778	0.99875
	BESAG (q = 1)	0.91013	0.875	0.911067	0.337468	0.99675
	BYM (q = 1)	0.981883	1	0.9814	0.672857	1
5	Getis Ord ${G}_{i}^{*}$	0.876948	0.035	0.961143	0.070917	0.908764
	Local Moran1	0.901364	0	0.9915	0	0.908372
	Local Moran2	0.903052	0.086429	0.984714	0.320417	0.915155
	SaTScan	0.990519	0.905714	0.999	0.990853	0.990706
	BESAG (q = 1)	0.953052	0.987143	0.949643	0.681104	0.998668
	BYM (q = 1)	0.95961	0.994286	0.956143	0.71302	0.999411
6	Getis Ord ${G}_{i}^{*}$	0.722013	0.341552	0.951875	0.807933	0.70567
	Local Moran1	0.751558	0.340345	1	1	0.715452
	Local Moran2	0.765	0.376897	0.999479	0.997831	0.726975
	SaTScan	0.954481	0.961552	0.950208	0.921588	0.976534
	BESAG (q = 1)	0.951104	1	0.921563	0.887697	1
	BYM (q = 1)	0.959091	0.999828	0.934479	0.904844	0.999894
7	Getis Ord ${G}_{i}^{*}$	0.464416	0.161771	0.965345	0.888718	0.410539
	Local Moran1	0.475584	0.15875	1	1	0.418321
	Local Moran2	0.505649	0.207083	0.999828	0.9995	0.43277
	SaTScan	0.818377	0.812813	0.827586	0.891381	0.752451
	BESAG (q = 1)	0.954221	1	0.878448	0.932773	1
	BYM (q = 1)	0.960714	1	0.89569	0.941899	1
Overall	Getis Ord ${G}_{i}^{*}$	0.806634	0.369753	0.960199	0.609452	0.813321
	Local Moran1	0.829654	0.355893	0.99308	0.666251	0.816561
	Local Moran2	0.833669	0.394446	0.987665	0.722482	0.822133
	SaTScan	0.953539	0.938231	0.954637	0.901673	0.952883
	BESAG (q = 1)	0.946061	0.977024	0.922771	0.75782	0.999236
	BYM (q = 1)	0.962684	0.998975	0.942165	0.826735	0.999869

Case scenarios 4 and 5 represented the situation with isolated hotspots (no large clusters) representing well-controlled or rare diseases. For these simulation scenarios, only SaTScan and Bayesian mapping could detect the hotspots with high accuracy, sensitivity and PPV in both cases as shown in Figures S4 and S5. In case scenario 4, Bayesian BYM mapping performed with 100% sensitivity, followed by SaTScan and Besag with 98.47% and 91.01% sensitivity respectively. For specificity, Bayesian BYM, SaTScan and Local Moran1 had the highest scores of more than 95%. For overall accuracy, Getis Ord ${G}_{i}^{*}$ had the lowest at 89.46% while SaTScan and Bayesian BYM yielded the highest with 98.47% and 98.18% respectively. In case scenario 5, Bayesian models had the best sensitivity while all methods yielded similar specificity. SaTScan had the best PPV whereas Bayesian models performed best on NPV. Getis Ord ${G}_{i}^{*}$ and Local Moran had the lowest sensitivity and PPV. Overall in this scenario case, SaTScan and Bayesian mapping had the best overall accuracy whereas Getis Ord ${G}_{i}^{*}$ performed worst.

**Fig. 2:**
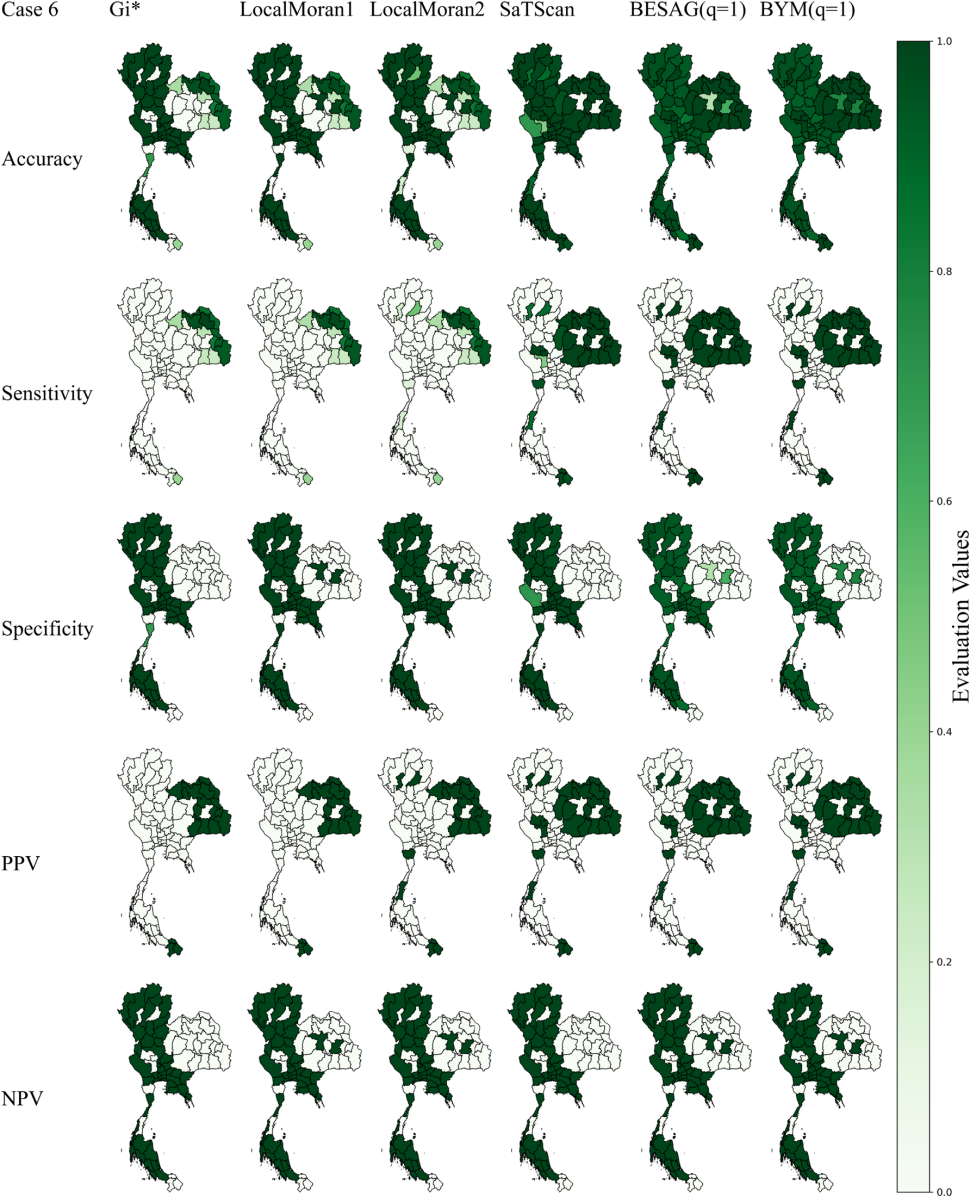
Maps of evaluation measures under simulated scenario 6

**Fig. 3:**
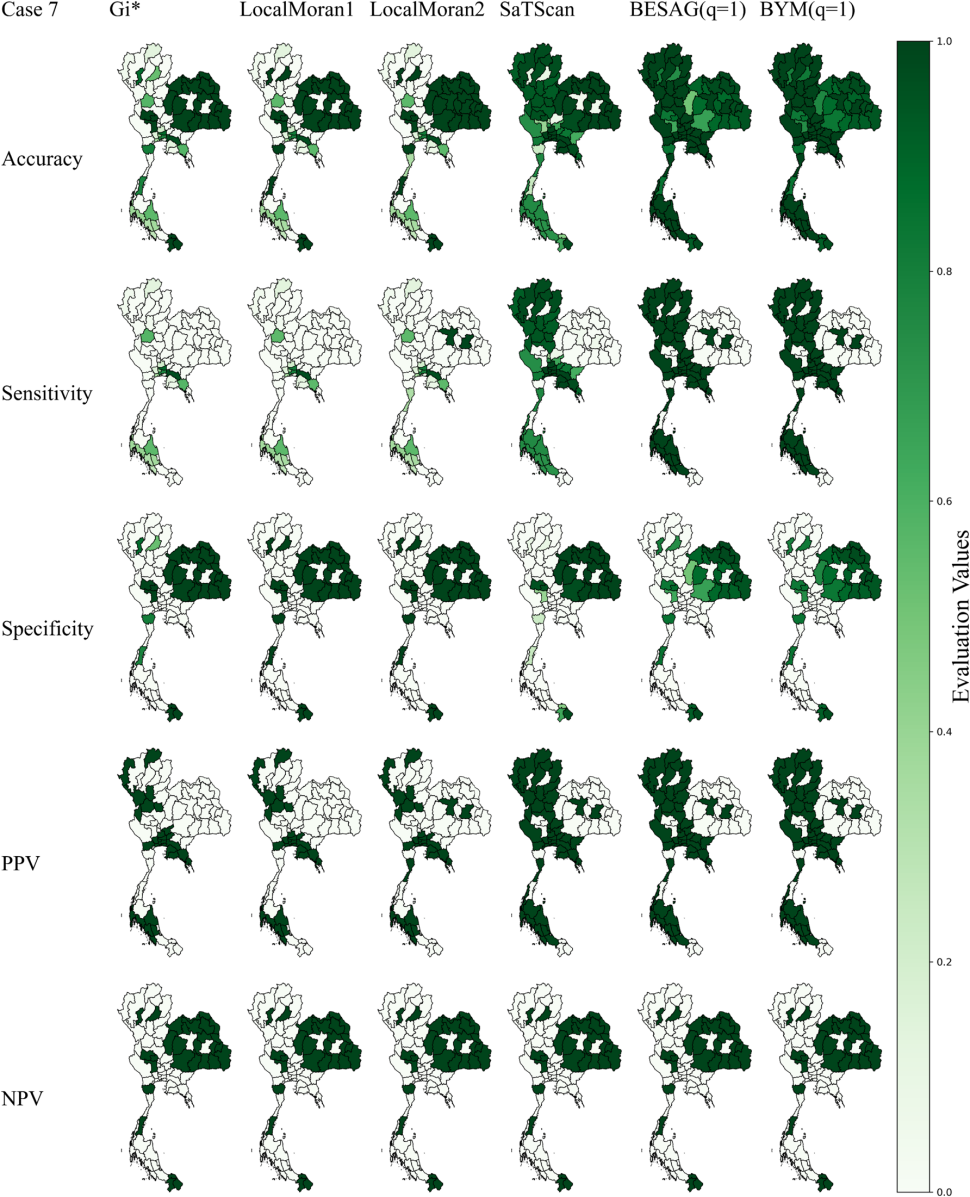
Maps of evaluation measures under simulated scenario 7

In case scenarios 6 and 7, the simulated clusters were combinations of scenarios 2–5 with different sizes and locations of spatial anomalies. These conditions should closely characterize real situations of various infectious diseases. Mapping results of case scenario 6 are depicted in Fig. 2. The Bayesian models still had the best sensitivity while SaTScan performed slightly worse. Interestingly, Getis Ord ${G}_{i}^{*}$ and Local Moran had much lower sensitivities, dropping to less than 40%, with their NPVs decreasing to less than 75%. Bayesian BYM had the best accuracy of 95.91% followed by SaTScan and Besag while the accuracies for Getis Ord ${G}_{i}^{*}$ and Local Moran were less than 80%. For case scenario 7, results are shown in Fig. 3. Bayesian mapping had the best sensitivity and NPV while Local Moran1 had the best specificity and PPV. Getis Ord ${G}_{i}^{*}$ and Local Moran still performed worst on sensitivity and NPV. The Bayesian BYM and Besag yielded the best accuracies of 96.07% and 95.42% respectively followed by SaTScan with 81.83% whereas Getis Ord ${G}_{i}^{*}$ and Local Moran poorly performed with overall accuracies of around 50%.

### Application to national dengue surveillance in Thailand

**Fig. 4:**
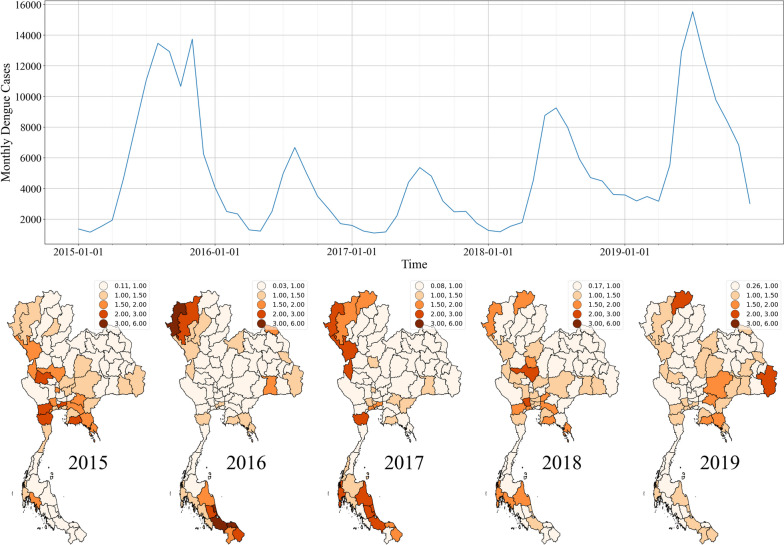
Plots of monthly dengue cases (top) and yearly provincial dengue SMR maps (bottom) during 2015–2019

Dengue presents a significant public health challenge in Southeast Asia, especially in regions burdened by high disease incidence and limited per capita healthcare resources. Thailand, as depicted in Fig. 4, grapples with a high number of annual dengue cases and recurrent outbreaks. This figure features a monthly observed total dengue case plot at the top and yearly provincial dengue SMR maps at the bottom, with expected rates computed using case data for each year from 2015 to 2019. This context highlights the nation's critical need for efficient resource allocation in disease control. Cluster detection methods play a pivotal role in facilitating decision-making by identifying high-risk areas for targeted interventions. While our simulation study shed light on cluster detection technique performance under various simulated scenarios, we now delve into a case study using real 2019 national dengue surveillance data from Thailand, aiming for a more comprehensive assessment of these methods. Within this case study, we explored different model specifications for Bayesian mapping, Local Moran's I, Getis Ord ${G}_{i}^{*}$ and SaTScan, seeking to assess their practical behavior and effectiveness in a real-world context.

**Fig. 5:**
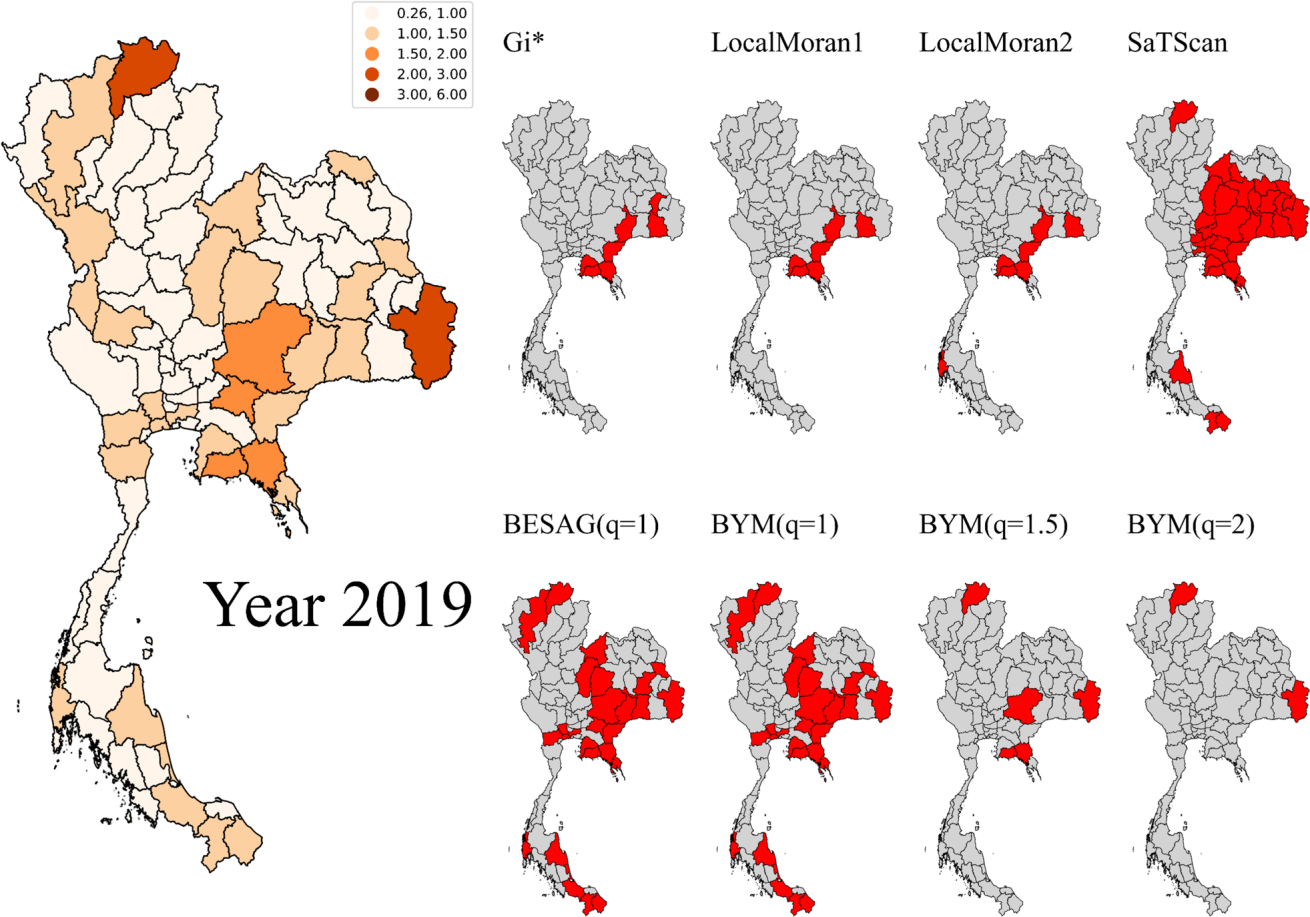
Maps of Thai provincial dengue SMRs in 2019 (left) compared with cluster detection results from different cluster detection methods (right). The significant hotspot provinces were labelled in red

Figure 5 shows the map of provincial dengue SMRs in 2019 in the left panel compared with cluster detection results from different cluster detection methods. The significant hotspots from each method are labelled in red. A clustered hotspot was identified in the central area using Besag and BYM while the others could not. Next, only some provinces in the east and lower part in the north east were declared as hotspots using Getis Ord ${G}_{i}^{*}$ and Local Moran while SaTScan recognized the whole eastern region and most of the northeast as a large cluster. Bayesian models with the cut-off threshold equal to the expected rate (*q* = *1*) detected only parts of the east and northeast as hotspots. Furthermore, isolated anomalies were notified by Bayesian methods while SaTScan mostly missed low-valued hotspots and only partly detected some in the north and south. For different threshold levels used in Bayesian models, clusters with SMR greater than the baseline were mostly declared as hotspots. In the cases specifying the threshold as 1.5 or 2 times larger than the expected rate (*q* = *1.5* and *2*), only provinces with extreme SMRs were alarmed. Overall, Bayesian models appeared to be the most sensible and compatible with the real dengue surveillance data in this case study.

## Discussion

Spatial surveillance employs diverse methodologies for the identification and characterization of disease clusters. In this study, we conducted a comprehensive evaluation of four widely employed cluster detection methods: Getis Ord ${G}_{i}^{*}$, Local Moran, SaTScan, and Bayesian disease mapping These methods, each characterized by unique specifications, exhibited notable variations in their performance. Our analysis included a series of simulation scenarios designed to emulate various infectious disease transmission situations, supplemented by empirical data from a real case study using national dengue surveillance.

### Performance variations in simulation study

In the context of our simulation study, we observed performance trends that shed light on the strengths and limitations of each method. Getis Ord ${G}_{i}^{*}$ and Local Moran demonstrated similar overall performance, possibly due to their shared computation formulas. This observation was consistent with prior research [23, 43] and aligns with the findings of Laohasiriwong's study [44]. Nevertheless, despite their comparable performance, these methods faced challenges in accurately defining cluster boundaries and identifying isolated hotspots. In contrast, SaTScan demonstrated robust overall performance, albeit with difficulties in detecting inner outliers, as also reported in Criag’s study [45].

Scenarios featuring isolated hotspots, such as those in scenarios 4 and 5, posed specific challenges for Getis Ord ${G}_{i}^{*}$ and Local Moran, resulting in a higher rate of false alarms. Conversely, SaTScan excelled in identifying isolated peaks. However, in scenario 6, Getis Ord ${G}_{i}^{*}$ and Local Moran struggled to partially detect anomalies in the northeast and missed an outbreak in the southern border. Further challenges arose in scenario 7, particularly in the western region for Getis Ord ${G}_{i}^{*}$ and Local Moran, though Local Moran2 effectively identified isolated peaks in the northeast. SaTScan, due to its window shape, faced limitations in detecting these isolated peaks, primarily due to its incompatibility with Thailand's irregular administrative boundaries [46].

Within the Bayesian models considered, most demonstrated an aptitude for detecting isolated areas. However, the Besag model exhibited slightly lower accuracy, likely attributable to the absence of an unstructured random effect. Conversely, introducing an unstructured random effect in the BYM model led to modest performance improvements in scenarios marked by spatial heterogeneity. The simulation results presented in Table 1 suggested the utilization of the Besag and BYM methods, positioning them as the preferred options. In contrast, Getis Ord ${G}_{i}^{*}$ and Local Moran, despite their extensive utilization in epidemiological research, delivered the least promising results across the spectrum of simulated scenarios.

### Empirical dengue surveillance data validation

The validation of these methods using real data from the Thai national dengue control program in 2019 confirmed distinct hotspot identifications. Besag and BYM effectively identified a clustered hotspot in the central region, a task unaccomplished by the other methods. Getis Ord ${G}_{i}^{*}$ and Local Moran primarily identified specific provinces in the east and the lower part of the northeast as hotspots, while SaTScan employed a distinct approach by designating an extensive region encompassing most of the east and northeast as a large cluster. Notably, the choice of threshold levels within Bayesian models significantly influenced hotspot identification, with larger thresholds selectively flagging provinces with extreme SMRs.

It is important to recognize that spatial health analyses traditionally concentrate on specific administrative regions, but the dynamic nature of geographic phenomena often transcends these predefined borders, introducing spatial edge effects and challenges related to censoring that can affect the performance of cluster detection methods [47]. In light of both our simulation study and empirical case study, Bayesian disease mapping, particularly through the BYM model, appeared as the optimal approach. This method demonstrated an adaptive capacity for detecting irregular disease anomalies and effectively addressed issues related to variance adjustments near boundaries. Furthermore, the flexibility offered by Bayesian models in defining clusters at varying baseline levels presents valuable options for shaping disease control strategies [48, 49].

### Limitations

We acknowledge certain limitations in the study. Firstly, SaTScan offers multiple configurations, including window shapes, circle size of the population at risk, and the size of the maximum population circle [38]. While we employed the standard default setting of SaTScan, future investigations should consider exploring alternative configurations. Additionally, since SaTScan is founded on the point process, its adoption should be carefully defined and aligned with the practicality of areal disease outcomes. Secondly, the Bayesian cluster detection in this study was based on a 95% exceedance probability threshold [50]. While this threshold holds practical value in public health investigations, it would be beneficial to examine different thresholds and baseline levels to optimize the method. Lastly, our study was primarily focused on spatial clusters, yet the interest of policy makers and public health professionals extends to cluster detection in both spatial and temporal dimensions.

### Public health implications and future directions

Our study offers valuable insights into the performance of cluster detection methods and highlights their potential for enhancing dengue surveillance. This information can assist public health agencies in endemic regions such as Thailand in responding more effectively to outbreaks, optimizing resource allocation, and improving public health outcomes. Furthermore, these methods can be adapted and generalized to the surveillance of other infectious diseases with similar transmission patterns, offering a broader framework for strengthening public health systems in response to emerging health threats.

While we consider our study a valuable addition to the disease mapping literature with the comparison of widely used cluster detection methods, we also see potential for broadening the scope of methods that can aid public health policymaking. Therefore, future research should expand into space–time cluster detection, entailing a comprehensive exploration of a wider range of clustering methods and more extensive investigations. This expanded research could encompass varying risk levels and scenarios, allowing for a more comprehensive analysis.

## Conclusions

Cluster detection is an important measure for infectious disease surveillance, especially within the context of the dengue control program in Thailand. Based on our simulations, the Bayesian modeling had the overall best performance for areal cluster detection followed by SaTScan which seemed mostly suitable for large clusters. The case study of dengue surveillance in Thailand provided similar results showing Bayesian disease mapping well captured spatial anomalies of different shapes and sizes while other methods seemed to effectively detect hotspots in limited circumstances. The evidence we have presented can enhance outbreak responses and assist authorities in the efficient allocation of resources for dengue surveillance in Thailand. Furthermore, the insights from our study are adaptable and have the potential for broader applications in the surveillance of infectious diseases with similar settings. Ultimately, this can empower authorities to make more informed public health decisions.

### Supplementary Information

**Additional file 1.**

## Data Availability

The case study dataset analyzed during the current study are publicly available on the surveillance reporting system website, Bureau of Epidemiology, Department of Disease Control, Thai Ministry of Public Health.

## Abbreviations

SMR: Standardized morbidity ratio
INLA: Integrated Nested Laplace Approximation
PPV: Positive predictive value
NPV: Negative predictive value
BYM: Besag-York- Molliè model
TP: True positive
FP: False positive
FN: False negative
TN: True negative
