# Supplementary material for: Evaluation and comparison of spatial cluster detection methods for improved decision making of disease surveillance: a case study of national dengue surveillance in Thailand
Source: BMC Med Res Methodol. 2024 Jan 19;24:14. doi: 10.1186/s12874-023-02135-9 (PMC10797994; doi:10.1186/s12874-023-02135-9)
Supplement: Supplementary file 1 — Additional file 1. [file 12874_2023_2135_MOESM1_ESM.docx]

**Evaluation and comparison of spatial cluster detection methods for improved decision making of disease surveillance: A case study of national dengue surveillance in Thailand**

**Supplementary document**

**S1 Spatial contiguity matrix**

The spatial contiguity matrix as an essential components for spatial models [1] was firstly described, then spatial clustering procedures including Getis Ord Gi*, Local Moran’s I coefficient, BYM, and Spatial Scan Statistics. Spatial contiguity matrices or spatial weight matrix is a square matrix that contains definition of spatial relationships for all node pairs, normally in the form of a zero-diagonal matrix including with off-diagonal non-zero values [2, 3]. Contiguity matrix construction for aggregated level is divided into 4 types related to the game of chess [4]. In this study, the queen contiguity criterion which binarily considered the consecutive neighbors for all eight directions [5] was chosen in accordance with provincial-aggregated data which was used in this study. The spatial weight matrix in this paper was formulated as

$$W=\left\{ w_{ij} \right\}$$

with

$$w_{ij}=\left\{ \begin{aligned} 1 ;pair\left( i,j \right) are reachable \\ 0 ;otherwise \end{aligned} \right.$$

$$i,j\in\left\{ n \right| 0\leq n<N; n,N\mathbb{\in N\}}$$

where $W$ is the spatial contiguity matrix that may include self-loop for all provinces, $w_{ij}$ is the spatial weight value for node pairs $(i,j)$ where $i,j$ are node indices for all $N$ provinces in systems which are elements in natural number. Matrix $W$ was applied for the spatial procedure in later steps.

**S2 Hypothesis testing of Anselin Local Moran’s I**

The hypothesis testing of local Moran’s I can be formulated as

$$H_{0}:I_{i}=0$$

$$H_{a}:I_{i}\neq0$$

which means spatial randomness is the null hypothesis. Significance level can be examined under Monte Carlo Randomization [6]. Monte Carlo Randomization for Local Moran’s I starts from simulating a set of spatial space $D_{k}^{'}$ which is a value re-localized version of original space $D$, then calculating Local Moran’s I on all spaces $D_{k}^{'}$ and defining these as distribution under $H_{0}$. A pseudo p-value at node $i$ ($\hat{p_{i}}$) is estimated as

$$\hat{p_{i}}=\frac{1+\sum_{k=1}^{K} f\left( i,k \right)}{K+1}$$

with

$$f\left( i,k \right)=\left\{ \begin{aligned} 1;I_{i,k}^{*}\geq I_{i} \\ 0;otherwise \end{aligned} \right.$$

$$k\in\left\{ 1\leq k\leq K;k,K\in\mathbb{I}^{+} \right\}$$

$$i\in\left\{ n \right| 0\leq n<N; n,N\mathbb{\in N\}}$$

where capital $K$ is the number of samplings, $I_{i}$ denotes Local Moran’s I at node $i$ on the original space $D$ while $I_{i,k}^{*}$ indicates the Local Moran’s I values at node position $i$ on all $k$ simulated spaces $D_{k}^{'}$, and $f\left( i,k \right)$ is a binary function with non-zero return whether statistics on the simulation space is more than the original space.

Moreover, the significance of Local Moran’s I can be classified using a Moran Scatter plot [7] which is quadrant-based segregation composed of 2 components, unit values or node values on the x-axis, and spatial lag on the y-axis which can be determined as

$$lag_{i}=\frac{\sum_{j=1}^{N} w_{ij}y_{j}}{\sum_{j=1}^{N} w_{ij}}$$

where $lag_{i}$ is the spatial lag value at node $i$, $N$ denotes count of nodes in spatial space, $y_{j}$ represents unit values or quantity at node $i$, and $w_{ij}$ indicates weight values in weight matrix $W$.

**S3 Maps of performance results of scenario cases 1-5 in the simulation study.**
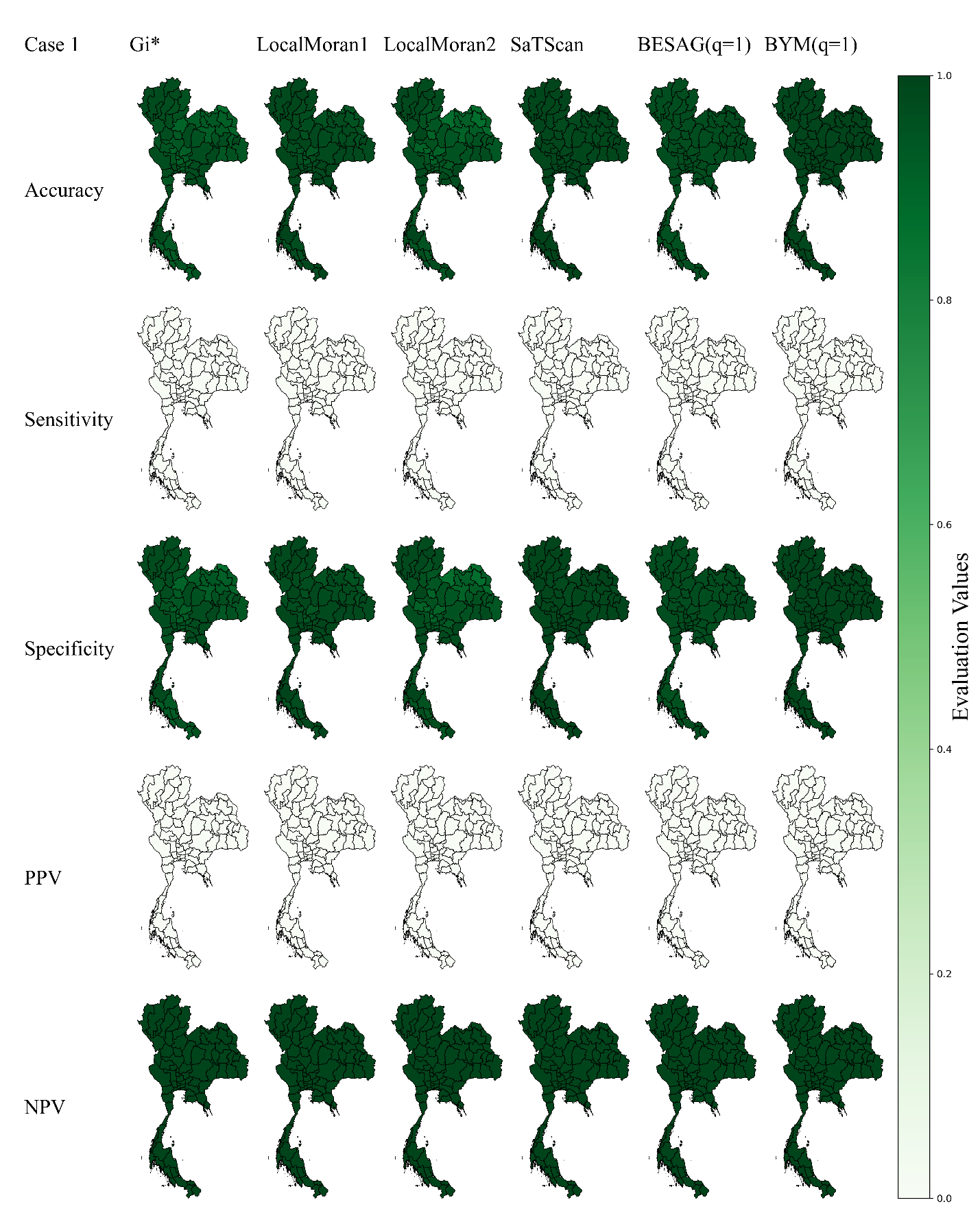


**Figure S1** Evaluation of interesting procedures for case 1, based on their accuracy, sensitivity, specificity, positive predictive value, and negative predictive value. The dark-colored areas indicate higher validation values.


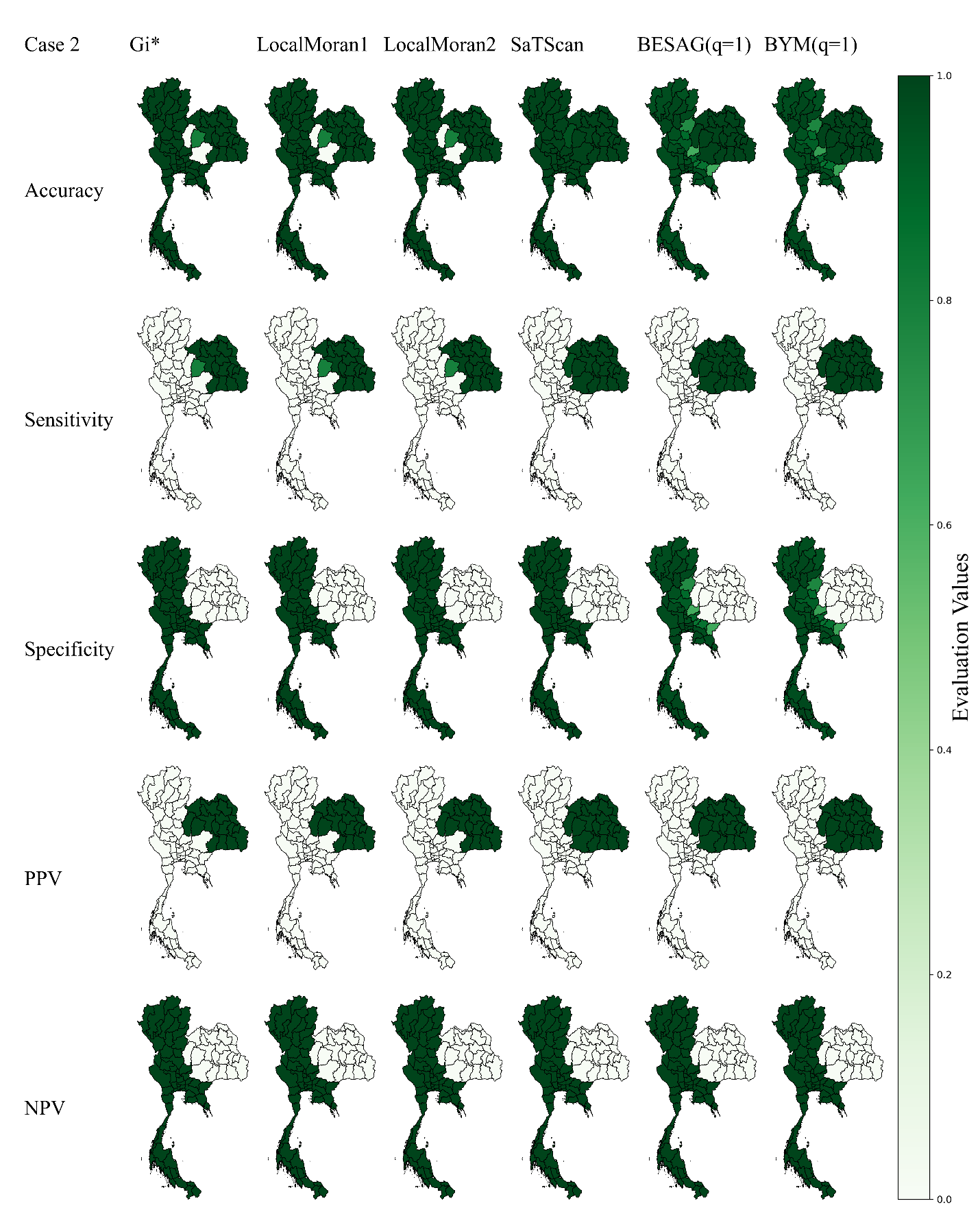


**Figure S2** Evaluation of interesting procedures for case 2, based on their accuracy, sensitivity, specificity, positive predictive value, and negative predictive value. The dark-colored areas indicate higher validation values.


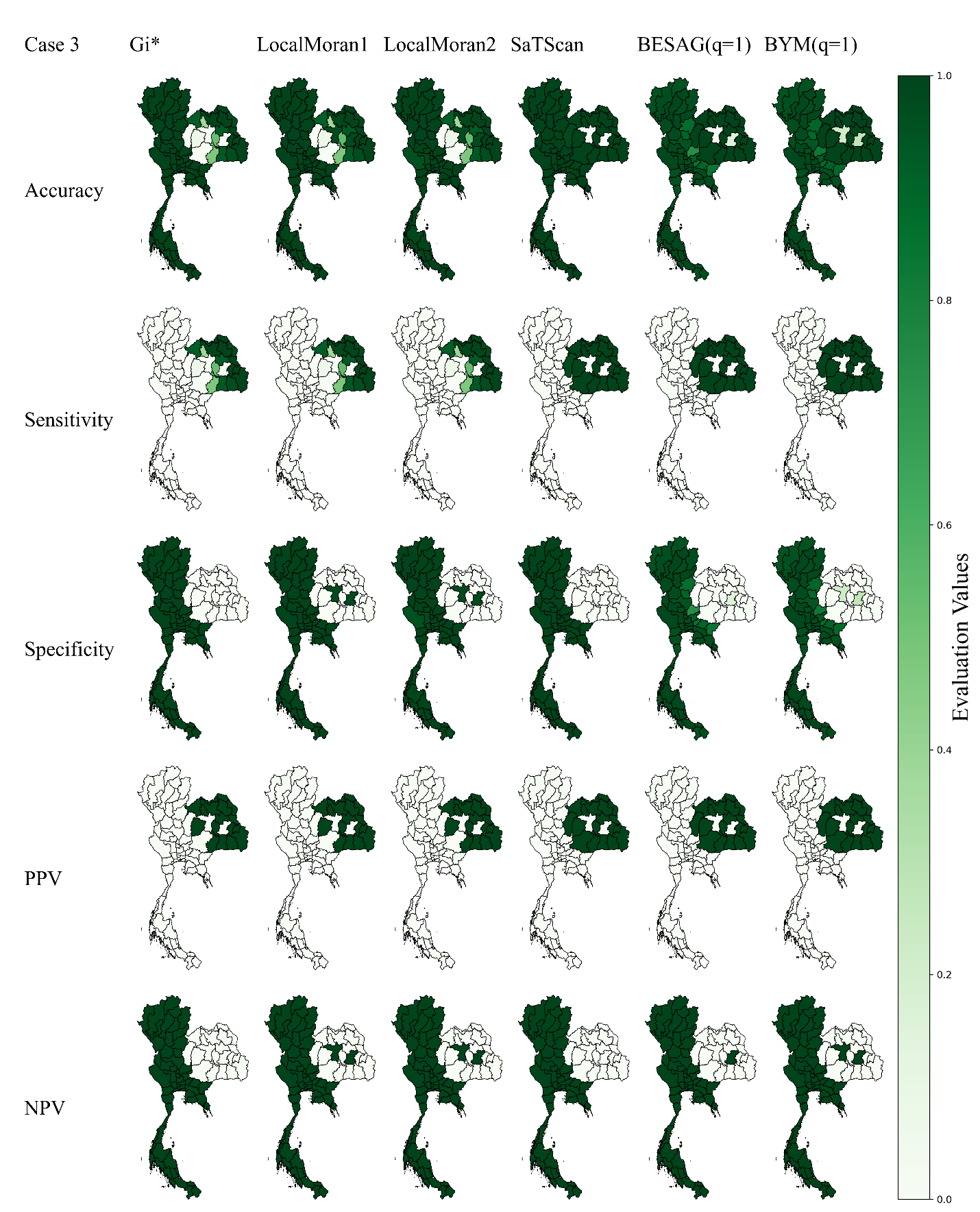


**Figure S3** Evaluation of interesting procedures for case 3, based on their accuracy, sensitivity, specificity, positive predictive value, and negative predictive value. The dark-colored areas indicate higher validation values.


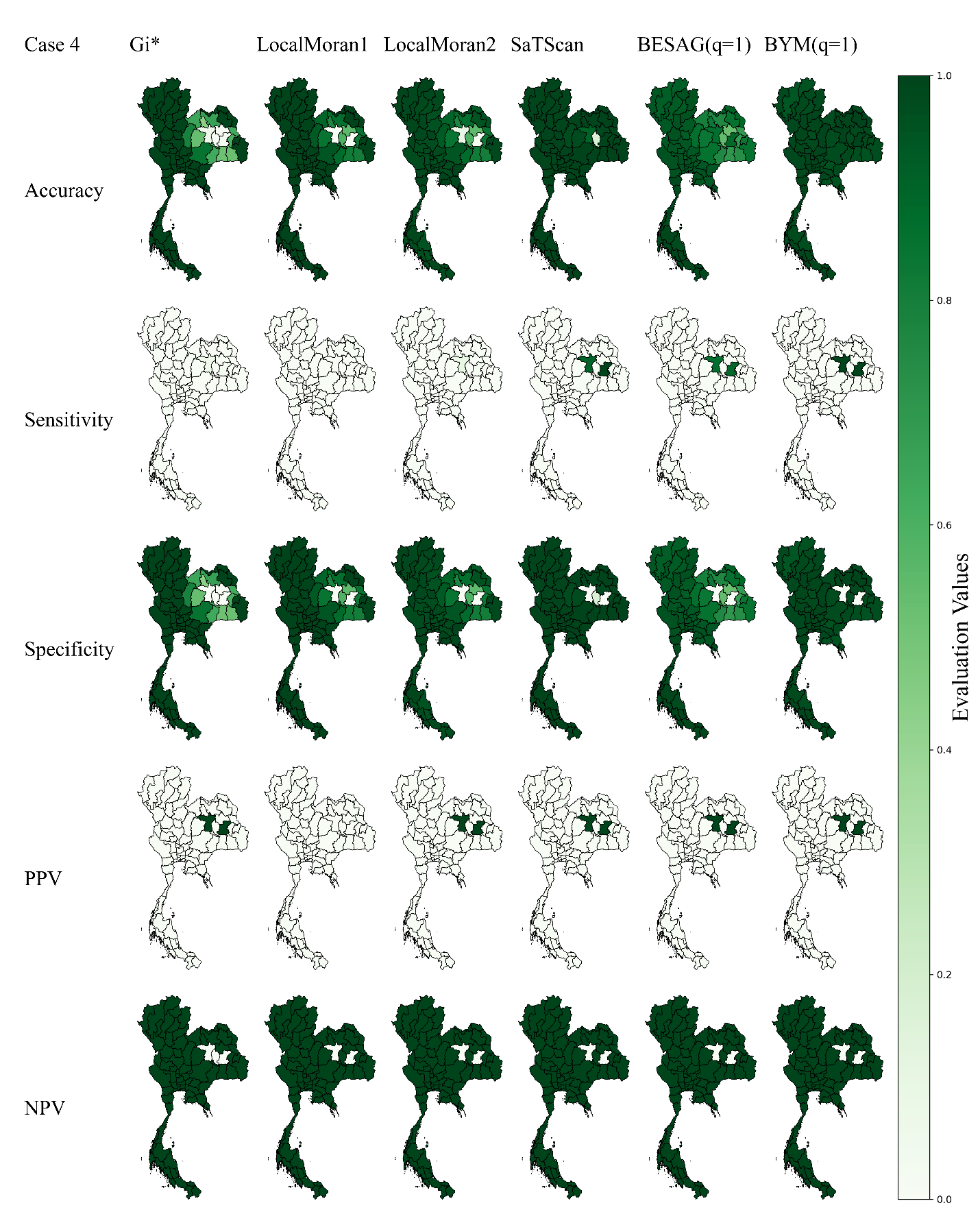


**Figure S4** Evaluation of interesting procedures for case 4, based on their accuracy, sensitivity, specificity, positive predictive value, and negative predictive value. The dark-colored areas indicate higher validation values.


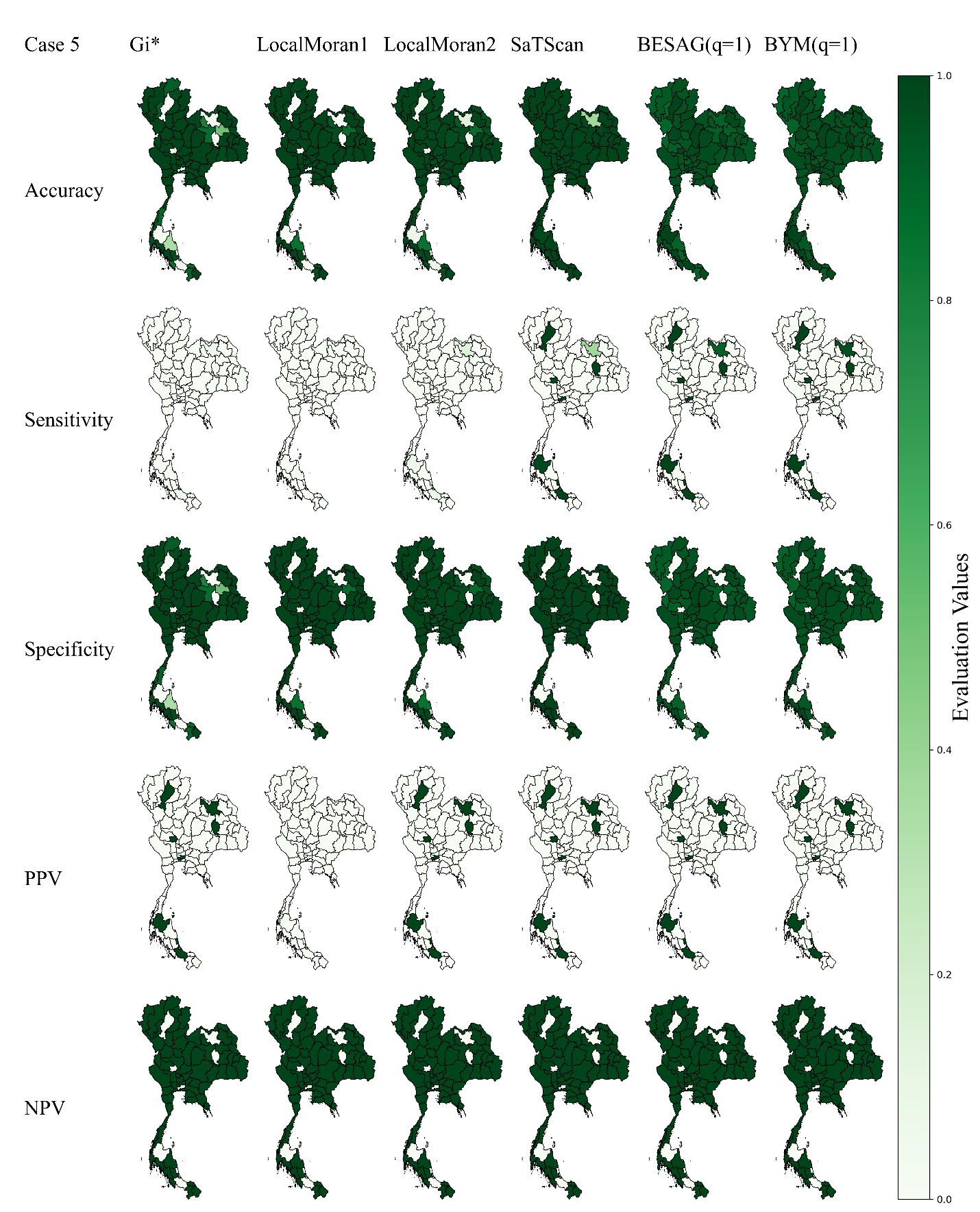


**Figure S5** Evaluation of interesting procedures for case 5, based on their accuracy, sensitivity, specificity, positive predictive value, and negative predictive value. The dark-colored areas indicate higher validation values.

**References**

1. Herrera Gómez, M., J. Mur Lacambra, and M. Ruiz Marín, *Selecting the most adequate spatial weighting matrix: A study on criteria.* 2012.

2. Sarrias, M., *Lecture 1: Introduction to Spatial Econometric.* Universidad Catolica del Norte, Chile, 2016.

3. Srinivasan, S., *Spatial Contiguity Matrices*, in *Encyclopedia of GIS*, S. Shekhar and H. Xiong, Editors. 2008, Springer US: Boston, MA. p. 1077-1078.

4. Kelejian, H. and G. Piras, *Chapter 1 - Spatial Models: Basic Issues**Basic texts in spatial analysis and econometrics are Cliff and Ord, 1973, Cliff and Ord, 1981, Anselin (1988), and Cressie (1993). More recent texts and compilations are Anselin and Florax (1995b), Anselin et al. (2004), Anselin and Rey (2014), LeSage and Pace, 2004, LeSage and Pace, 2009, Elhorst (2014), and Arbia, 2006, Arbia, 2014. See also a nice overview of spatial models, and the development of spatial econometrics by Anselin, 2002, Anselin, 2009 and Anselin and Bera (1998)*, in *Spatial Econometrics*, H. Kelejian and G. Piras, Editors. 2017, Academic Press. p. 1-10.

5. Getis, A. and J. Aldstadt, *Constructing the Spatial Weights Matrix Using a Local Statistic.* Geographical Analysis, 2004. **36**(2): p. 90-104.

6. Sarrias, M., *Lecture 1: Introduction to Spatial Econometrics.* Chile: Universidad de Talca, 2020.

7. Aldstadt, J., *Spatial Clustering*, in *Handbook of Applied Spatial Analysis: Software Tools, Methods and Applications*, M.M. Fischer and A. Getis, Editors. 2010, Springer Berlin Heidelberg: Berlin, Heidelberg. p. 279-300.
